# Surface Fouling Characterization Methods for Polymeric Membranes Using a Short Experimental Study

**DOI:** 10.3390/polym16152124

**Published:** 2024-07-25

**Authors:** Numan Yanar, Shinyun Park, Eunmok Yang, Heechul Choi

**Affiliations:** 1 R&D Center, NAiEEL Technology, 6-2 Yuseongdaero 1205, Daejeon 34104, Republic of Korea; 2 Department of Civil and Environmental Engineering, Colorado State University, Fort Collins, CO 80523, USA; shinyun.park@colostate.edu; 3 School of Earth Sciences and Environmental Engineering, Gwangju Institute of Science and Technology (GIST), 261 Cheomdangwagi-ro, Buk-gu, Gwangju 61005, Republic of Korea; yang1990@gm.gist.ac.kr

**Keywords:** SEM, CLSM, AFM, WLI, membrane characterization, membrane fouling

## Abstract

Membrane surface fouling has always been a critical issue for the long-term operation of polymeric membranes. Therefore, it is crucial to develop new approaches to prevent fouling. While developing new approaches, characterization methods are greatly important for understanding the distribution of fouling on the membrane surface. In this work, a cellulose acetate membrane was fouled by the filtration of artificial wastewater based on alginate. The surfaces of fouled membranes were characterized through scanning electron microscopy (SEM), confocal laser scanning microscopy (CLSM), atomic force microscopy (AFM), and white light interferometry (WLI). The results were then compared in terms of the resolution, accuracy, feasibility, and cost-efficiency.

## 1. Introduction

For many people, water security—the ability to maintain sustainable access to reliable supplies of water of acceptable quality—is already under jeopardy, and things will become worse over the next few decades [1,2]. Therefore, there is a requirement for the development of advanced water treatment methods to meet the rising demand for clean and safe water [3]. Related to this, wastewater treatment and desalination are becoming critically important for the utilization or reuse of available water supplies [4,5,6,7,8]. Currently, many methods are commonly used for this purpose, such as thermal-based separations and membrane separations. Among these, the competitiveness of membrane technology for the treatment of wastewater and water in recent decades has been encouraging, and membrane separations are at the forefront as they have better performance in terms of energy consumption and cost [9,10,11]. This technology employs high-quality to low-quality water from brackish water, sea water, surface water, and well water supplies [12,13,14,15]. Membranes are categorized as either organic or inorganic based on the composition of their constituent materials. While inorganic materials such as ceramics, metals, zeolites, or silica are used to make inorganic membranes, organic polymers such as synthetic polyethylene (PE), polytetrafluorethylene (PTFE), polypropylene (PP), cellulose acetate (CA), and cellulosetriacetate (CTA) are used to create organic membranes [16,17]. These polymeric membranes are used mostly for pressure-driven separation techniques, such as forward osmosis (FO) (an osmotic pressure-driven process), microfiltration (MF), ultrafiltration (UF), nanofiltration (NF), and reverse osmosis (RO) [18,19,20,21]. Although these separation methods are preferable in terms of their high selectivity and energy efficiency, there are numerous significant drawbacks to polymeric membranes. These include fouling caused by their innate hydrophobicity and surface charge, the degradation of membranes in salt/wastewater interactions due to their cationic (Na^+^, K^+^, Mg^2+^, etc.) reaction with electron-rich functional groups (OH, COOH, CO, etc.) in polymers, and the decomposition of membranes at higher temperatures due to random crosslinking and depolymerization reactions. Due to these drawbacks, polymeric membranes display incompatibility issues with the present membrane market since there is a significant trade-off between the performance and lifetime of the membranes [22,23]. Among the drawbacks of polymeric membranes, the inevitable phenomenon of membrane fouling is a critical obstacle that must be addressed [24,25,26].

Membrane fouling is a very complicated issue that is yet not fully understood. It is induced by the deposition and adsorption of microscopic particles or macromolecules (internal fouling) within the membrane’s pores or by the clogging of membrane pores by cells, cell debris, organics, colloids, and particles on the membrane surface. Numerous forms of fouling, such as inorganic fouling or scaling, organic fouling, biofouling, and colloidal fouling, can occur in membrane systems depending on the membrane process and chemical composition of the foulants [27,28,29]. To decrease or prevent fouling, characterization methods are of great importance, as inaccurate results can lead to erroneous research conclusions. Currently, various microscopic or topographic methods are widely used to characterize membrane fouling, such as scanning electron microscopy (SEM), scanning tunneling microscopy (STM), transmission electron microscopy (TEM), atomic force microscopy (AFM), Rutherford backscattering spectrometry (RBS), white light interferometry (WLI), confocal laser scanning microscopy (CLSM), and quartz crystal microbalancing (QCM) [30,31,32,33,34,35,36,37,38,39,40]. These test methods characterize the surface fouling layer’s many morphological, physical, chemical, and biological characteristics [41]. Among them, SEM, AFM, CLSM, and WLI are the most commonly used.

After the invention of AFM, a Nobel prize-winning characterization method, fouling research began employing this method since it is capable of obtaining topographic data from which foulant roughness on membrane surfaces can be analyzed visually. Although AFM can be considered an efficient method for understanding the roughness of fouled membrane surfaces, it is not simple as it entails some issues that must be handled. Considering how membrane fouling can be rough depending on the fouling material, AFM may not be an accurate method for detecting foulant thickness; however, it is an efficient method for detecting surface roughness at the nanoscale [42]. Modern CLSM was introduced after AFM [43]. However, it took time to apply this technology for the characterization of membrane fouling. Ferrando et al. utilized CLSM for the first time for membrane fouling characterization after filtrating BSA–fluorescein and ovalbumin–Texas red conjugates on an ultrafiltration membrane [44]. Later on, this method was further employed by various research groups as it is convenient for visualizing and detecting the thickness of the foulant on surfaces as well [45,46,47]. Even though AFM and CLSM are widely used for fouling characterization, SEM is also attractive for many researchers as it is easily accessible as a result of its long history and its capability for visualizing foulants for fouling characterization [48,49,50,51,52]. Additionally, WLI, which, like AFM, also employs a topographic approach, is another convenient method for characterizing membrane fouling. WLI is also a simple and cost-effective method that can provide topographic images of fouled membrane surfaces. Even though it is similar to AFM in terms of the obtained topographic data, its working mechanism is much more simple. It is also capable of obtaining data from larger surface areas with lower precision. All four of these methods are considered efficient for detecting the foulant thickness and average roughness or for visualizing and distributing foulants on membrane surfaces. Each method, however, has its own advantages and disadvantages. Therefore, it is crucial to understand the efficacy of each method based on experimental data. In this article, we compared SEM, AFM, CLSM, and WLI in terms of their convenience and reliability for fouling detection. This article carries great importance by being the first of its type as it aims to encourage both senior and early-career scientists to develop their skills for membrane fouling characterization.

## 2. Methods and Materials

### 2.1. Preparation of Artificial Wastewater

For the fouling operation, we prepared an artificial wastewater solution that has previously been demonstrated to accelerate alginate fouling by 3 times compared to solely using alginate [53] (Table 1).

### 2.2. Preparation of Filtration System

A cellulose triacetate (CTA) forward osmosis (FO) membrane from Hydration Technologies Innovation (HTI, USA) was used. Due to its asymmetric and rough surface structure, fouling is more concentrated on the membrane surface [54]. Furthermore, due to the high mechanical strength of CTA membranes, any possible deformation during the fouling or characterization process can also be avoided. Membranes were cut to a size of 4.5 cm × 5.5 cm, which is the size of the membrane cell as well. Alginate fouling was carried out for 400 min with an engineered osmosis system with a 0.6 M NaCl solution at the draw side and artificial wastewater at the feed side. After the fouling was completed, the membrane was oven-dried for 24 h at 40 °C and cut into small pieces for the characterization.

### 2.3. Characterizations

AFM (XE-100, Park Systems, Suwon, Republic of Korea), low-vacuum scanning electron microscopy (LV-SEM) (Jeol JP/JSM-6610LY, Tokyo, Japan), CLSM (Leica CTR 6500, Wetzlar, Germany), and WLI with a 3D optical profiler (3D-OP) (Nanoview NV-E1000, Ansan, Republic of Korea) were used for characterizations. Furthermore, field emission scanning electron microscopy (FESEM) (Zeiss Gemini 500, Jena, Germany) and energy-dispersive spectroscopy (EDS) (Oxford Instruments NanoAnalysis, Wycombe, UK) were also used in combination with an elemental and chemical analysis of fouled surfaces for a short EDS study on the supporting material.

By using SEM imaging, images of fouled and pristine membranes, zoomed in by 200× through LV-SEM, and 5000× through FESEM, were obtained. In addition, EDS analysis was also performed through the FESEM system to map regions with particular atoms in nanostructures.

For CLSM characterization, it was necessary to stain the fouled and unfouled membrane. A total of 5 mg of concanavalin A (Alexa Fluor 633 Conjugate, Thermo Fisher Scientific, Waltham, MA, USA) and 1 mL of a NaHCO_3_ (0.1 M, pH 8.3) buffer were mixed to prepare a stock solution. Later, 200 µL of the stock solution was diluted with 1800 µL of the NaHCO_3_ buffer (0.1 M, pH 8.3). For drying the membranes, they were kept in a dark room for 30 min after 200 µL of the solution had been poured onto them. As unbounded stains were used, we used a 1 mM CaCl_2_ solution to remove residues. Next, the samples were placed in 500 µL of a 1 mM CaCl_2_ solution for 5 min 3 times, and stained membranes were placed between cover glasses by mounting the membrane surfaces with 15 µL of ProLong Gold Antifade Mountant (Thermo Fisher Scientific) to make a smoother surface for characterization. After the mounting process, the membranes with cover glasses were firmly stuck and were subsequently kept in a dark room for 12 h to dry before characterization. Images of 5 Z-stack fields for fouled and nonfouled samples were taken by CLSM characterization [46,47]. For the visualization of the samples, Imaris x64 (Bitplane, Schlieren, Switzerland) was used.

The AFM study was conducted for the characterization of 45 μm (x-y) scan sizes of the samples cut to a size of 9 mm^2^ for the characterization of 5 μm × 5 μm areas. A non-contact cantilever with an XY scanning range of 50 µm and a Z range of 12 µm was used for the measurements and for the calculation of average roughness (R_a_), root mean square roughness (R_q_) values, and height (R_z_) values.

Finally, WLI was used through the 3D-OP with a vertical scan range of 180 μm, a horizontal scan range of 65 μm~650 μm with a horizontal resolution of 0.2 to 4 um, and a vertical resolution of 0.5 nm via white light scanning interferometry (WSI) and 0.1 nm via phase shift interferometry (PSI). Samples were cut to the size of 25 mm^2^, and images zoomed in by 10× and 50× were taken. Similar to AFM, average roughness (R_a_) and root mean square roughness (R_q_) values were obtained. This method provided us with topographic values in a way similar to AFM. Differently from AFM, however, 3D-OP uses reflected infrared light for the topographic imaging process.

## 3. Results and Discussion

In this section, the use of SEM, CLSM, AFM, and WLI for the surface fouling characterization of membranes is discussed.

SEM works by imparting kinetic energy to generate signals based on the interaction of electrons. To examine crystalline elements and photons, SEM uses secondary electrons, backscattered electrons, and diffracted backscattered electrons. While the backscattered electrons display a contrast in the composition of the specimen’s elements, the secondary electrons emitted by the specimen identify the morphology and topography of the specimen [55].

**Figure 1:**
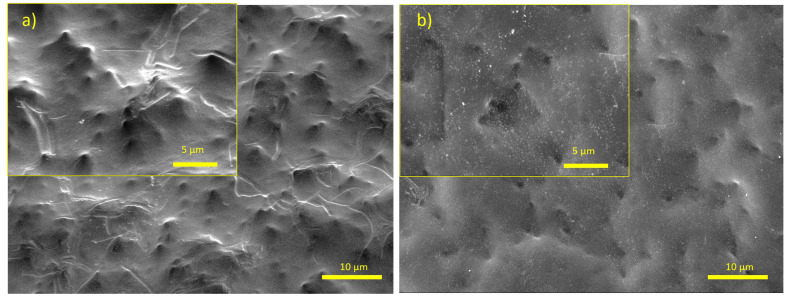
SEM images of membranes zoomed 2000× and 5000×: (**a**) image of an unfouled membrane; (**b**) image of a fouled membrane.

**Figure 2:**
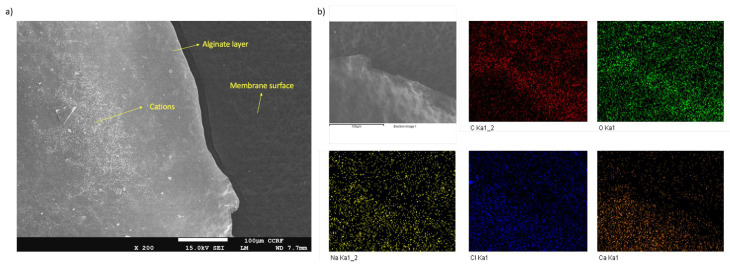
(**a**) SEM image showing an alginate layer and cations on the membrane surface; (**b**) EDS mapping of the fouled surface for different elements.

Regarding membrane fouling characterization, SEM is capable of accurately characterizing the existence, type, and aggregation of foulants on membrane surfaces. Through SEM, it is also possible to characterize the surface blocking of membranes. However, the measurement of the foulant volume or thickness should be considered at a large scale by taking the average volume and thicknesses of various areas. This is because the thicknesses of the fouling layers change in every point, and it is not possible to acquire exact values in all x-y-z axes. For our case, a thin layer of alginate is clearly visible in the images in Figure 1. Comparatively, a fouled membrane has a lighter skin, and the aggregation of foulants in some parts is also visible. In some parts, thick alginate layers can also be observed. In order to better investigate the fouling of such thick layers, an SEM image of the alginate layer on the membrane surface was taken at 200× zoom after scratching some parts of the fouled layer. Here, the edge lines of the alginate can clearly be seen together with the calcium ions concentrated in the alginate layer (Figure 2a), similar to the indicated structures in previous studies [56]. SEM is also advantageous when coupled with EDS mapping since the existence of specific elements on the fouled layer can be detected through mapping (Figure 2b). This is very crucial especially for detecting organic and inorganic foulants as in the given case of alginate fouling. SEM/EDS is a combination method for material analysis that uses an energy-dispersive X-ray spectrophotometer and a scanning electron microscope. SEM performs the imaging, whereas EDS handles the detection. Although most types of EDS are not capable of detecting ‘light’ elements with an atomic number of less than 10, elements from atomic number 4 (Be) to 92 (U) could potentially be observed.

Unlike SEM, CLSM can provide more detailed information about the volume distribution and thickness of the foulant. The capacity to serially make thin (500 nm to 1.5 micrometer) optical layers through fluorescent specimens with a thickness of up to 50 μm or more is the main benefit of CLSM. The picture series is created by coordinating consecutive image acquisition at each stage with incremental adjustments to the microscope’s fine focus mechanism. Due to decreased background fluorescence and enhanced signal-to-noise, the contrast and definition are noticeably high for wide-field surface fouling observations. Additionally, optical sectioning removes the artifacts that are present when tissue samples are physically sectioned and fluorescently stained for conventional types of microscopy. The non-invasive confocal optical sectioning technique makes it possible to examine the foulant with greater clarity and under a range of circumstances. This is even more useful for biofouling detection, including microorganism detection [57].

**Figure 3:**
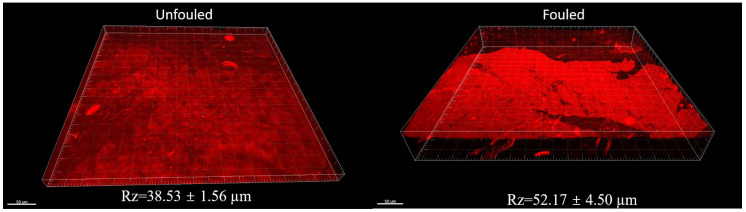
CLSM images of unfouled and fouled samples with R_z_ values.

For our case, the CLSM images clearly showed the amount of fouling on the membrane surface through an analysis employing Imaris x64 software. The height of the foulant layer from any point of the membrane surface was easily obtained. Furthermore, obtaining the foulant volume in a specific area was also possible. Even though CLSM does not provide the chemical characterization of the foulant, it is very efficient for excluding the membrane surface from the foulant. Our study showed that even unfouled membranes show a layer through CLSM characterization. Therefore, the approximate average thickness of the fouling layer can be calculated by taking the layer thickness difference between the fouled and unfouled surfaces. For our case, the unfouled membrane showed a 38.53 ± 1.56 µm layer thickness, while the fouled membrane showed a 52.17 ± 4.50 µm layer thickness (Figure 3). Here, the difference can be used to calculate the approximate value of the foulant thickness on the membrane surface.

Although CLSM offers various advantages, such as a high resolution, the digitization of the images, or its capability to include thin optical sections on different planes [58], it has some disadvantages as well. To start with, including thin optical sections can be useful for other applications of CLSM; however, it is actually a disadvantage for membrane fouling characterization since some parts of the surface layer of the membrane are also included in the foulant thickness data. Second, the operating cost of CLSM is very high. There are just a few excitation wavelengths that can be used with conventional lasers, and they are all costly to manufacture in the UV area and can occur across extremely small bands [57]. The prices of the equipment, software, and chemical materials used for prefabrication are also relatively high compared to in other techniques. Furthermore, the preparation of the samples also takes more time than for the other foulant characterization approaches.

**Figure 4:**
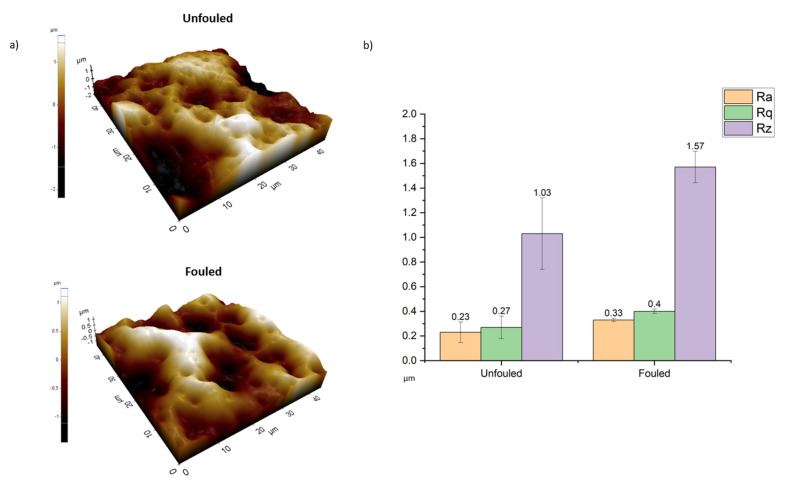
(**a**) Topographic images of unfouled and fouled samples from AFM; (**b**) R_a_, R_q_, and R_z_ values of the surfaces of unfouled and fouled membranes.

AFM scans the sample surface using a cantilever and a pointed probe. The forces between the probe’s tip and the sample cause the cantilever to deflect when it approaches a surface. Then, surface topography and phase maps are produced by the adjustment of the distance between the tip and the sample through a feedback mechanism. By obtaining data on the relative smoothness of a surface’s profile through AFM, the surface roughness can be calculated [59,60]. AFM is a great technique in terms of its accuracy at the nanoscale. However, membrane fouling can create even millimeter-scale roughness. Some research suggests that AFM can also be used for fouling thickness detection as well for the analysis of R_z_ values obtained by taking the nonfouled part of the sample as a minimum height. However, at this point, it is not possible to detect the nonfouled part, as it is a nanoscale measurement. Furthermore, the scanning size of AFM is very limited. Indeed, the biggest limitation of AFM is its characterization range, which is limited to imaging objects with a maximum height of 10–20 μm and a maximum scanning area of 150 µm × 150 µm [61]. Furthermore, another issue with AFM measurements is related to the piezoelectricity of the materials, although this was not an issue for our sample [61]. Hysteresis of the piezoelectric material and cross-talk between the x-, y-, and z-axis can also have an impact on AFM pictures, which may need software filtering and augmentation. However, such filtering may flatten the topographical characteristics. It should be noted that this issue was eliminated in recently invented AFM models. Considering the alginate fouling in our case, piezoelectricity was not an issue. However, in our case, foulant filtration took a long time, easily passing the scanning range limit of AFM. For the equipment that we used, we were able to scan a 45 µm × 45 µm area in one scan. From such a scan area of our samples, we calculated the R_a_ (average roughness), R_q_ (root mean square value of roughness), and R_z_ (height) values from different 5 µm × 5 µm point areas (Figure 4). The fouled membrane can be seen to be relatively rough; however, this is an indicator of nanoscale roughness and does not reflect the microscale roughness. It is known from previous research in the literature that when a surface becomes fouled, the nano-roughness values increase in the same ratio [62]. Therefore, for fouling detection purposes, AFM can only be considered as an interpretive tool, not as a means to obtain numerical values for the amount of foulant on the surface. It should also be noted it is very crucial in AFM to select the correct probe and scanner type. Most of the advanced AFM equipment utilizes different types of scanners depending on the scanning range based on the peaks and the valleys on the membrane surface. In the case of probe selection, the membrane and fouling materials are very crucial. For instance, in the case of the organic fouling of a polymeric membrane, silicon-based probes can successfully operate. However, in the case of strong materials, as in the case of inorganic foulants, advanced probe options should be investigated further.

**Figure 5:**
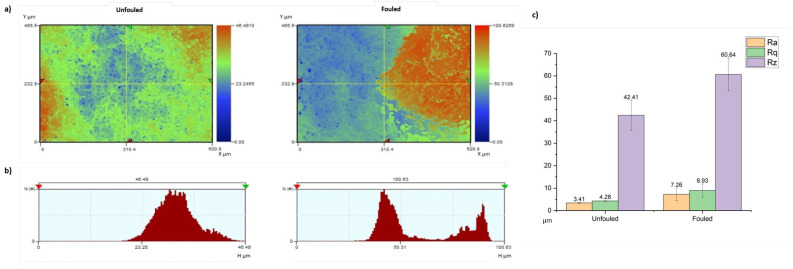
(**a**) Topographic images of unfouled and fouled samples from WLI; (**b**) Z axis view (2D) of unfouled and fouled samples (**c**) R_a_, R_q_, and R_z_ values of the surfaces of unfouled and fouled membranes.

Finally, WLI was used for the characterization of the fouling. In WLI, a light source emits white light and is separated into two beams by a beam splitter. The reference beam is sent to the reference surface, behaving as a mirror, while the sample beam is directly sent to the surface of the sample. The reference mirror reflects the passing beam to the couple charged device (CCD) image sensor, creating an interference pattern. The other beam forms an image at the CCD image sensor after being divided by a beam splitter and being reflected off the sample surface. Then, the CCD camera digitizes the interferograms, and a software is used to turn them into a topographic map [63]. However, WLI has a critical disadvantage when it is used for fouling detection. Even though it is capable of measuring surfaces as small as on the sub-nanometer scale, it can only detect reflecting surfaces. Thus, polymer membranes with a dark-colored foulant on their surface or possessing a dark-colored additive that changes the membranes’ color cannot be successfully measured by WLI. This reflection problem is not limited to just dark surfaces. Reflection can also be a problem if the surface has rough and curvy areas at the nanoscale. This can be better understood when our samples are reviewed. There is clear foulant deposition on the fouled sample. The R_a_ (average roughness), R_q_ (root mean square value of roughness), and R_z_ (height) values also show that the fouled sample has a rougher surface with a higher average surface height. The nonfouled sample has an average R_z_ value of 42.41 µm, while it is 60.64 µm for the fouled sample (Figure 5). That is, the difference between these samples can give an idea about the average foulant thickness on the surface. However, there is a critical error in this calculation, which is caused by the unreflected surface parts. When the surface images are seen, the small circular shapes on the surfaces are areas that are not reflected due to the bumpy shapes at the nano- and microscale (Figure 5a). Moreover, there is also a resolution issue for the WLI measurements. The limited number of sample data sets results in a low resolution for the XY stage measurements (approximately 300,000). The maximum number of data sets that certain white light interferometry devices can employ is 980,000. Furthermore, possible vibrations during the measurement can also create measurement errors. Therefore, setups should be installed on shock-adsorbing tables [64].

It should also be noted that the calculation of the membrane foulant roughness through AFM and WLI plays an important role in understanding fouling acceleration since roughly deposited surfaces are known to accelerate further fouling due to the enhanced surface area.

Among all four methods, AFM and SEM excel in terms of their precision. However, the scanning range of the measurements is very limited for AFM, and SEM is not capable of generating topographic data. CLSM and WLI can yield more useful results in terms of the fouling thickness and data stability. However, the difficult and costly process of CLSM characterization makes it less accessible for most researchers. Therefore, WLI appears to be the most suitable method in terms of its scanning range, effectiveness and low cost. However, its limitations for tortuous or non-reflecting surfaces make it less convenient for many studies. For more detailed information, see Table 2.

## 4. Conclusions

In this article, SEM, AFM, CLSM, and WLI methods were discussed in terms of their application to and working mechanism for characterization. Furthermore, the methods were analyzed in terms of their accuracy, efficacy, feasibility, and cost-effectiveness. It can be concluded that even though SEM is still the most accurate method for visualizing surface fouling, it alone is not as sufficient as CLSM or WLI for obtaining information about the fouling thickness. On the other hand, AFM was found to be inconvenient for analyzing microscale fouling due to its limited scanning size. When the CLSM and WLI methods are compared, it can be said that the R_Z_ value differences between the unfouled and fouled samples obtained from both equipment were very similar. Therefore, even though both CLSM and WLI have some disadvantages, such as the expensive costs of CLSM or the light reflection problem of WLI, they can be considered to be convenient and accurate methods for obtaining data about the fouling thickness through R_z_ value differences. Depending on the research budget or the membrane material, they can also be considered for combined use with SEM image visualizations of the foulant. For further research, these characterization methods should be considered in terms of their capacity to analyze fouling on the surfaces of inorganic membranes. In addition, nanomaterial-blended polymeric membranes should also be considered for future characterization research.

## Figures and Tables

**Table 1:** Materials used for preparing an artificial wastewater solution.

Material	Concentration	Type
Sodium alginate	75 mg/L	Sigma Aldrich (St. Louis, MO, USA), CAS: 9005-38-3
Ammonium chloride	0.94 mM	Sigma Aldrich, CAS: 12125-02-9
Potassium phosphate, monobasic	0.45 mM	Oriental Chemical Industry (Cranford, NJ, USA), CAS: 7778-77-0
Calcium chloride dihydrate	0.50 mM	Duksan (Ansan, Republic of Korea), CAS:10035-048
Sodium bicarbonate	0.50 mM	DC Chemical Co. Ltd. (Shanghai, China), CAS:144-55-8
Sodium chloride	2.00 mM	Sigma Aldrich, CAS: 7647-14-5
Magnesium sulfate	0.60 mM	Oriental Chemical Industry, CAS: 7487-88-9

**Table 2:** Comparison table for SEM, AFM, CLSM, and WLI for their working principles and other parameters [31,65,66,67,68].

	SEM	AFM	CLSM	WLI
Principle	Detect intensity of emitted secondary electron	Measure forces between probe and surface	Detect emitted fluorescence signals from a single point of focus	Detect light interference occurring in the distance traveled by white light
Resolution	0.5–1 nm	~1 nm	Lateral: ~140 nm Vertical: ~1 µm	Lateral: ~1 nm Vertical: ~160 nm
Vertical scanning range	Not applicable	Up to 20 µm	Up to 500 µm	Up to 20 mm
Sample preparation	Dry and metallic coating steps	Dry step	Proper fluorescent stain and dry steps	Dry step
Challenges for membrane fouling characterization	− Potential charging issue for non-conductive samples	− Limited application for samples with differences in height	− Limited imaging depth − Limited application to foulants that can be stained	Low accuracy for extremely rough, tortuous, or non-reflecting surfaces
Destructive /Non-destructive	Dynamic destructive	Static non-destructive	Destructive	Static non-destructive
Relative cost	Medium	Medium	High	Low

## Data Availability

Data are contained within the article.
